# Functional diversity of habitat formers declines scale-dependently across an environmental stress gradient

**DOI:** 10.1007/s00442-020-04746-1

**Published:** 2020-09-07

**Authors:** Laura Cappelatti, Alizée R. L. Mauffrey, John N. Griffin

**Affiliations:** Biosciences Department, Swansea University, Wallace Building, Swansea, SA2 8PP Wales UK

**Keywords:** Emersion gradient, Seaweed, Functional traits, Emergent groups, Assembly patterns

## Abstract

Marine habitat formers such as seaweeds and corals are lynchpins of coastal ecosystems, but their functional diversity and how it varies with scale and context remains poorly studied. Here, we investigate the functional diversity of seaweed assemblages across the rocky intertidal stress gradient at large (zones) and small (quadrat) scales. We quantified complementary metrics of emergent group richness, functional richness (functional space occupied) and functional dispersion (trait complementarity of dominant species). With increasing shore height, under species loss and turnover, responses of functional diversity were scale- and metric-dependent. At the large scale, functional richness contracted while—notwithstanding a decline in redundancy—emergent group richness and functional dispersion were both invariant. At the small scale, all measures declined, with the strongest responses evident for functional and emergent group richness. Comparisons of observed versus expected values based on null models revealed that functional richness and dispersion were greater than expected in the low shore but converged with expected values higher on the shore. These results show that functional diversity of assemblages of marine habitat formers can be especially responsive to environmental stress gradients at small scales and for richness measures. Furthermore, niche-based processes at the small—neighbourhood—scale can favour co-occurrence of functionally distinctive species under low, but not high, stress, magnifying differences in functional diversity across environmental gradients. As assemblages of marine habitat formers face accelerating environmental change, further studies examining multiple aspects of functional diversity are needed to elucidate patterns, processes, and ecosystem consequences of community (dis-)assembly across diverse groups.

**Electronic supplementary material:**

The online version of this article (10.1007/s00442-020-04746-1) contains supplementary material, which is available to authorized users.

## Introduction

Biodiversity is increasingly recognised as multidimensional, including taxonomic, phylogenetic, and functional components (Cadotte et al. 2009; Devictor et al. 2010; Stevens and Gavilanez 2015). Functional traits such as body size, mouthpart- or leaf- morphology determine an organism’s capacity to process resources and how it experiences and interacts with its environment. The diversity of such traits—functional diversity—captures variation in the ecological roles of species (Tilman 2001; Violle et al. 2007; Díaz et al. 2020). Although ecologists have traditionally focused on taxonomy-based metrics of biodiversity, incorporating functional diversity allows a more complete view of how communities respond to environmental gradients and human pressures (Mouillot et al. 2013; Teixidó et al. 2018; Sol et al. 2020; Muguerza et al. 2020), elucidates the relationships between biodiversity and ecosystem functioning (e.g. Griffin et al. 2009; Lefcheck and Duffy 2015), and reveals the operation of niche-based processes during community assembly (Mcgill et al. 2006). Functional diversity approaches have been widely tested and applied in terrestrial ecosystems structured by primary producers and habitat formers such as trees, grasses, and climbing plants (Roderick et al. 2000; Mokany and Ash 2008; Seger et al. 2017). There is now growing interest in applying functional diversity approaches to assemblages of habitat formers in marine and coastal ecosystems as they rapidly reorganise under global change (Blowes et al. 2019; Antão et al. 2020).

Habitat-forming organisms such as corals, seagrasses, and seaweeds form diverse assemblages and support associated biodiversity and ecosystem functioning in many coastal environments worldwide (Stachowicz et al. 2007; Thomsen et al. 2010; Teagle et al. 2017; Vergés et al. 2019). In temperate nearshore systems, macroalgae (seaweeds) often dominate standing biomass and contribute to services from carbon sequestration to coastal defence and fisheries support (Smale et al. 2013). Global changes including ocean warming and acidification, invasive non-native species and coastal urbanisation are driving marked shifts in seaweed assemblages (Wernberg et al. 2011; Harley et al. 2012). Functional approaches to seaweed assemblages predominantly involve grouping species based on position in the canopy (e.g. Tait et al. 2014) or general morphology (e.g. Steneck and Dethier 1994). Yet the functionality of species and assemblages is determined by multiple, often continuous, traits, challenging coarse a priori grouping (Chapin et al. 1996; Fong and Fong 2014; Mauffrey et al. 2020a). Here, we use the rocky intertidal as a test case for applying a suite of continuous traits to seaweeds to understand patterns of functional diversity across environmental gradients.

Multiple, complementary, metrics are required to describe how functional diversity changes along gradients of disturbance or stress (Villéger et al. 2008; Mouillot et al. 2013). Functional diversity can be quantitatively defined as the distribution of species in a functional space where the axes represent combinations of traits (Rosenfeld 2002; Mason et al. 2005; Villéger et al. 2008). Metrics used to describe functional diversity include functional richness—the total extent of species in trait space—and functional dispersion—the distinctness of dominant species (Laliberté and Legendre 2010). Functional richness and dispersion indicate the potential for species complementarity, respectively emphasising differences between extreme and abundant species (Mason et al. 2005; Kuebbing et al. 2018). Although declines in species richness along environmental gradients are expected to erode redundancy and particularly affect functional richness, these changes can be buffered in systems with high redundancy (Micheli and Halpern 2005; but see Mouillot et al. 2014). Contrarily, strong environmental constraints on the viable traits (environmental filtering) can drive greater-than-expected declines in functional richness (Mouillot et al. 2013; Valdivia et al. 2017; Teixidó et al. 2018) and may reduce the dispersion of dominant species (Schellenberger Costa et al. 2017). Investigating changes in multiple facets of functional diversity across environmental gradients can, therefore, add to the traditional taxonomic focus and provide a complementary lens to understand community structure and potential consequences for ecosystem functioning (Díaz and Cabido 2001; Zhang et al. 2012).

The rocky intertidal has long served as a proving ground for ecological ideas due to its accessible marine communities and provides a model system to examine functional diversity of seaweeds along an environmental stress gradient. The intertidal rocky shore is characterised by a vertical pattern of community turnover (or ‘zonation’) and decreasing species richness. These community changes are driven by an interplay between biotic interactions and species’ tolerances to tidal emersion period and associated stressors of desiccation, temperature extremes and nutrient scarcity (e.g. Schonbeck and Norton 1979; Underwood and Jernakoff 1984; Scrosati and Heaven 2007). The intertidal stress gradient can be mitigated by rock pools which create habitat heterogeneity on beds of emergent rock and commonly host different suites of less desiccation-tolerant species (Araújo et al. 2006; Firth et al. 2014). The rocky shore and attendant community gradients remain a classic research and teaching system in ecology and are prominent in contemporary ecological textbooks (Begon et al. 1996; Singer 2016). Zonation patterns in seaweed communities have been studied in rocky shores in several parts of the world: from the North Atlantic (Lubchenco 1980; Dring and Brown 1982) to high latitudes of Alaska and the Magellanic region (Ingólfsson 2005). However, beyond studies of the traits of dominant species (Gómez and Huovinen 2011; Cappelatti et al. 2019), the corresponding responses of functional diversity in these assemblages remain poorly appreciated.

Here, we revisit the classical pattern of intertidal seaweed zonation from a functional perspective. With information on community composition and species’ traits across four rocky shores in Wales (UK), we investigated the functional structure of seaweed assemblages along the emersion gradient (shore height). First, we hypothesised that the increasing stress towards the upper intertidal would act as an environmental filter, constraining viable trait values and resulting in a disproportionate loss of functional diversity relative to species loss. Second, we hypothesised that a turnover in species composition would lead to a functional turnover in communities, as trait values reflect different adaptations to the upper shore environment. To address these hypotheses, we examined changes in diversity across the shore height gradient at the scale of entire zones (total species lists) and local communities (replicate quadrats). We further investigated the role of rock pools in modifying diversity gradients via habitat heterogeneity and explored functional redundancy across zones by assigning species to clusters in trait space (i.e. emergent groups).

## Materials and methods

### Study sites and survey

We studied macroalgal communities along the intertidal gradient across four rocky shores in the Gower peninsula, south Wales (UK). The carboniferous limestone shores span ca. 25 km, and have a tidal variation of approximately 10.4 m: Oxwich (sheltered; 51.55 N, 4.15 W), Bracelet Bay (semi-exposed; 51.57 N, 3.98 W), Overton (exposed; 51.53 N, 4.21 W) and Rhossili (exposed; 51.56 N, 4.32 W).

Surveys were conducted during the summer of 2018, by identifying and quantifying all living macroalgal species within 1 × 1 m quadrats, placed 10 m apart along four transects. Each transect crossed the whole intertidal from the kelp zone in the lower shore to the end of seaweed distribution in the upper shore. We sampled the low shore at low tides only during spring tides (0–0.5 m above chart datum). Cover was estimated within quadrats with the help of strings which divided the quadrat into 25 sub-units (each unit = 4% cover). Individuals were thoroughly manipulated to ensure inclusion of small, understory seaweeds, therefore, the multi-layered communities could surpass 100% cover.

**Fig. 1:**
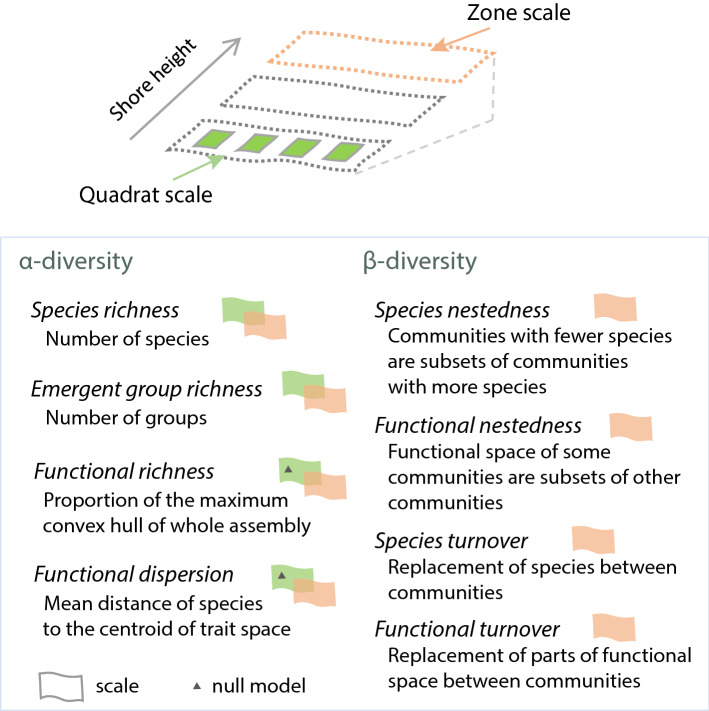
Conceptual illustration of study scales and description of diversity metrics. Flags next to each metric indicate at which scale they were measured: in green, small scale (quadrat) and in orange, large scale (zone). Triangles indicate cases where we also compared observed to null values

To address the intertidal emersion gradient, we used commonly defined zones of the low, middle, and upper shore (Fig. 1). In agreement with previous studies (Stephenson and Stephenson 1949; Ballantine 1961; Johnson et al. 1998; Chappuis et al. 2014), these zones were defined by their relative position in the intertidal combined with the identity of dominant Phaeophytes (fucoids and kelp). Specifically, zones were delimited where the following species alone or together comprised in excess of 80% of the total macroalgal cover: low shore—*Laminaria digitata* and *Fucus serratus*; mid-shore—*Ascophyllum nodosum and F. vesiculosus*; upper shore—*Pelvetia canaliculata* and *F. spiralis*. To obtain a clearer distinction between zones, we excluded quadrats falling in the transition between zones as defined above; accordingly, zones were separated by at least 20 m. Although zone serves as an indicator of shore height, and thus emersion period, the exact position of these zones along shore height gradients depends on additional factors such as wave exposure and disturbance history (Ballantine 1961). These three main zones are thus conceptualised as ‘community types’ positioned sequentially with respect to shore height rather than communities occurring at precise shore heights. In our study, the characteristic fucoids or kelps in each zone comprise a minority of the assemblage (low shore: 5%; mid-shore: 6%; upper shore: 11%) and our findings are robust to exclusion of these species (Appendix 1, ESM). Details on the sampling effort across zones are in Appendix 2, ESM.

### Habitats

We distinguish between two simple habitat types on the rocky shore: emergent rock and rock pool. During the survey, we recorded the percentage cover of these two habitat types. Preliminary analyses revealed that the distribution of rock pool cover was highly uneven, with half of the quadrats having zero cover. Furthermore, the relationship between rock pool cover on seaweed species richness was complex and mostly realised within the first 5% of cover. Accordingly, for simplicity, we treat rock pools as a binary presence (≥ 5% cover) or absence (< 5%) variable. Quadrats that landed at least partially on gullies or deep rock pools (> 50 cm depth) were not considered, since the projected area would be much larger than the other quadrats.

After sampling all shores, we randomly selected a subset of quadrats to obtain an equal representation of combinations of shore zone (low, middle, and upper shore) and habitat (presence/absence of rock pools). The final analysed community data comprised 84 quadrats, 14 for each zone and habitat category combination. Of the selected quadrats, 15 were from Bracelet Bay, and 22, 23, and 24 from the remaining sites.

### Trait data

Traits were obtained from a database in development since 2016 (see Mauffrey et al. 2020b). For the creation of this trait database, we conducted frequent summer sampling trips predominantly in south Wales; all but two of the species in this study were sampled for trait screening from the same sites as the survey data used here. We collected a minimum of 3 individuals per species (mean and mode = 6, max = 45). This wide range in replicates is due to the varying abundance of species, and to our efforts to sample abundant species at different sites to capture their intraspecific variation (Cappelatti et al. 2019). To obtain comparable traits, we restricted our study to erect macroalgae only (i.e. not encrusting forms).

The traits we chose are “functional markers” (sensu Garnier et al. 2004) and thus are proxies for physiological or physical functions. We summarise the traits in Table 1 and describe methods for their collection as well as ecological relevance in Appendix 3 (ESM). Prior to analyses, all traits were log-transformed and scaled to zero mean and unit variance, to approach normal distributions and have the same range of variation. Traits were always measured at the species level; however, some species require laboratory identification, so they were only identified at the genus level. In the case of *Ulva*, this genus was further divided into two groups related to obvious phenotypic differences. For the artificial taxa “green sheet”, we used trait averages from *U. linza* and *U. lactuca*; for “green tubular” we used trait averages from *U. intestinalis* and *U. compressa*.

**Table 1:** Summary of traits used in this study

Eco- physiological aspect	Ecological significance	Trait and unit	Related mechanism	Weight
Photosynthetic ability	Investment in photosynthesising structure maximises light capture, increasing productivity. Trade-off: lower investment in structural compounds affects integrity^a,b^	Specific thallus area—STA (mm^2^ g^−1^)	High STA increases the proportion of light-absorbing surface^c^	0.33
		Surface area to volume ratio -SA:V (mm^2^ mL^−1^)	High SA:V increases the proportion of nutrient- and gas- exchange surface^b^	0.33
		Thickness (mm)	Thicker fronds allow survival and draught resistance^a,d^	0.33
Structure	High ratios of hard structural to soft palatable tissue protects against desiccation and increases recalcitrance, contributing to carbon storage. Trade-off: slows productivity^h^	Thallus dry matter content—TDMC (ratio)	High TDMC confers structural integrity^e,f^	0.50
		Carbon to Nitrogen ratio—C:N	High C:N confers structural integrity^e,g^	0.50
Space use	Exploration of space, linked to competitive dominance, resource acquisition and habitat provision^i^. Trade-off: potentially increases water drag^j^	Length (cm)	Holds seaweed high in water column, facilitates access to light^a,b^	0.25
		Pneumatocysts (yes/no)	Holds seaweed upwards in water column, facilitates access to light^k^	0.25
		Branching order (integer)	Increases complexity, may maximise light exposure^l^ and delays desiccation^m,n^	0.25
		Surface area to perimeter ratio—SA:P	Reversely related to branching order; higher SA:P decreases complexity	0.25

Traits are divided into three groups, according to the eco-physiological aspects of macroalgae and the respective ecological significance. Proposed mechanisms linking traits to ecophysiology are provided. Each trait was assigned a weight for functional diversity metrics so that each of the three aspects of eco-physiology were equally represented

^a^Carpenter (1990)

^b^Littler and Littler (1980)

^c^Roderick et al. (2000)

^d^Reich et al. (1999)

^e^Cornelissen et al. (2003)

^f^Elger and Willby (2003)

^g^Weykam et al. (1996)

^h^Rodriguez et al. (2016)

^i^Steneck and Dethier (1994)

^j^Starko et al. (2015)

^k^Dromgoole (1981)

^l^Stewart and Carpenter (2003)

^m^Hay (1981)

^n^Taylor and Hay (1984)

### Trait space of species

**Fig. 2:**
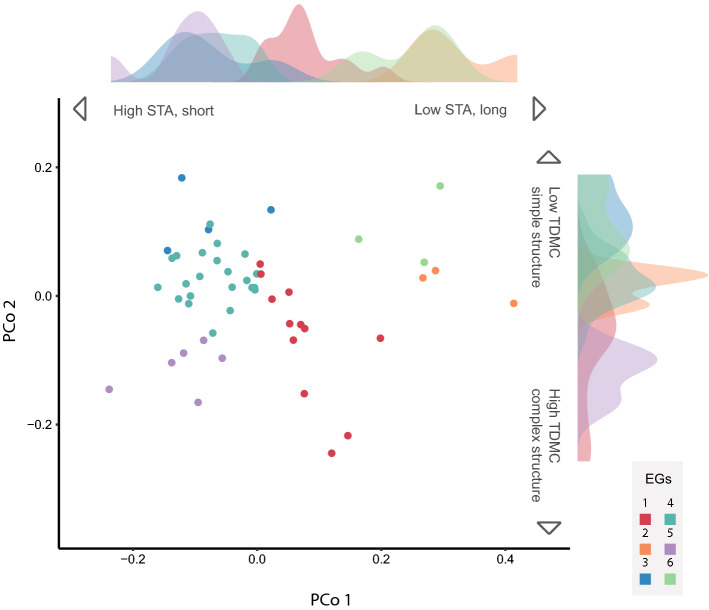
Principal Coordinates of study species based on 9 traits. Each point represents a species (*n* = 50) and is coloured by the emergent group. Emergent groups (EGs) were created from a trait-based Gower distance and, therefore, may not be fully represented in this two-dimensional plot. Density plots show the distribution of emergent groups along each PCo axis. Arrows summarise correlations between traits and axes

Species’ functional trait differences were characterised based on a Gower distance matrix, at the whole study scale. Traits were weighted to ensure that all three aspects of functionality considered here (photosynthesis, physical structure, complexity) were equally represented (Table 1). To visualise species’ functional diversity as captured in the Gower distance matrix, we used principal coordinate analysis (PCoA) and displayed the first two axes, which explained 61% of inertia (Fig. 2; see Appendix 3, ESM, for further details of the quality of functional space representation).

To examine species’ contributions to functional diversity and redundancy across zones, we also addressed species’ occupancy of parts of the trait space via emergent groups. Because emergent groups are based on measured traits, they should be a more accurate representation of species’ differences than the traditional functional groups (Mauffrey et al. 2020a). In our study communities, emergent groups allowed us to observe redundancy (species within groups share similar trait values) and how parts of the trait space (as occupied by different groups) change across zones. Species were grouped based on the Gower distance matrix, using the *k*-medoids clustering method (Reynolds et al. 2006) applied with *pam* in package *cluster* (Maechler et al. 2019). We tested increasing numbers of groups and selected the highest number of groups (i.e. 6) where all pairwise contrasts using PERMANOVA were significant at *α* = 0.05 (Hervé 2020; Mauffrey et al. 2020a).

### Diversity metrics

We calculated species and functional diversity metrics at both the large (zone) and small (quadrat) scales (Fig. 1). The larger-scale includes all quadrats within a zone across the four study sites and provides the most complete estimate of the species occupying the specific zone in the area/region. This scale of diversity includes large-scale heterogeneity across sites and represents species that may contribute to ecosystem functionality over larger spatial and temporal scales (Isbell et al. 2017). The smaller scale of the 1 × 1 m quadrat (the local or “neighbourhood” scale) represents the scale at which direct biotic interactions occur between seaweeds (e.g. Edwards and Connell 2012). Seaweeds respond to the stresses associated with shore height at both scales, but community composition also varies at the quadrat scale due to differences in local factors such as the presence of rock pools.

We calculated four complementary α-diversity metrics: species richness, emergent group richness, functional richness, and functional dispersion (Fig. 1). Species and emergent group richness are simply the total number of species or emergent groups present at a focal scale. While species richness is a standard metric of biodiversity in rocky shore assemblages (e.g. Scrosati et al. 2011) and treats all species equally, emergent group richness is based on species’ relative positions in trait space and only declines when entire regions of trait space (emergent groups) have been vacated. Functional richness is the size of the functional space filled by the community, based on the convex hull delimited by the most extreme points in functional space (Cornwell et al. 2006; Villéger et al. 2008). We scaled functional richness to a hypothetical community with all species in the study pool, to benchmark values relative to the potential maximum. Functional dispersion is the mean abundance-weighted distance in multidimensional space of individual species to their overall centroid, thus measuring species’ distribution in trait space (Laliberté and Legendre 2010). To calculate functional richness and dispersion, we selected PCoA axes from the Gower distance matrix among communities (details on the PCoA are in Appendix 3, ESM). As the above-described metrics quantify diversity for individual communities, they are measures of α-diversity. All α-diversity indices were calculated using function *dbFD* on package *FD* (Laliberté and Legendre 2010).

Patterns of β-diversity reveal underlying changes in community structure along environmental gradients (Legendre 2014). While taxonomic β-diversity captures the changes in the identities of the species, functional β-diversity captures changes in the locations of species in trait space. To examine β-diversity across zones, we used the Sorensen index, which measures the ratio of shared species (or overlapping volume of convex hull) among assemblages to the mean number of species (or mean convex hull volume) occurring in a single assemblage (Gotelli and Chao 2013). The returned index is a measure of the multiple site dissimilarity among communities (Baselga 2010). At both species and functional levels, β-diversity was partitioned between nestedness and turnover components (Fig. 1). These indices were calculated with functions* beta.multi* (species incidences) and *functional.beta.multi* (based on the PCoA) in package *Betapart* (Baselga and Orme 2012).

### Statistical analyses

To investigate if the observed functional diversity metrics of local—small scale—communities were lower or higher than expected based on the number of species within a community, we used a null model approach. Null communities (999 in each case) were constructed by shuffling species’ position on the trait matrix, thus holding constant the overall functional structure of seaweeds and species richness within each observed community (quadrat), while randomizing the identities of species in each null community (Swenson 2014). We then calculated the standardized effect sizes (SES) of functional richness and functional dispersion, which compares observed to null values (Cadotte and Tucker 2017). For each focal aspect of functional diversity (richness, divergence) and each community (*n* = 84) a single SES value was generated. We used one-sample *t* tests (or Wilcoxon rank tests, when data distribution was not normal) to compare observed SES values against null SES values.

We used linear models to investigate the additive and interacting effect of shore height (zone) and habitat heterogeneity (presence/absence of rock pool) on species and functional α-diversity metrics, and on SES values. We also examined the observed α-diversity metrics across sites and found no significant differences in their means; the site was then dropped from the analysis as our focus was on general effects of zone (Appendix 4, ESM).

## Results

### Species distribution in the trait space

Fifty taxa (referred to as species hereafter) were found in the survey: 43 identified at the species level, and seven at the genus level (including the two artificial groups of *Ulva*). The majority (*n* = 33) were red algae (phylum Rhodophyta), followed by brown algae (*n* = 10, class Phaeophyceae) and green algae (*n* = 7, phylum Chlorophyta).

The first PCo axis was most strongly related to traits linked to photosynthesis: species with higher PCo 1 values tended to display reduced specific thallus area (STA), reduced surface area to volume ratio (SA:V) and have thicker thalli (Fig. 2; Table 1). The second axis mainly related to traits representing space use and structure: species with higher PCo 2 values tended to have lower thallus dry matter content (TDMC) and branching order, and higher surface area to perimeter ratio (SA:P). Generally, PCo 1 translates into a trade-off between resource acquisition and conservation (i.e. an “economic” trade-off) and PCo 2 into a complexity / structural gradient. All trait-axis and trait-trait correlations are shown in Appendix 3, ESM. Overall, species were distributed unevenly across the functional space and, therefore, emergent groups had varying numbers of species (from 3 to 22, Figs. 2 and 4). The large richness observed within groups 1 and 4 shows they hold a high degree of redundancy.

### Large scale (zone level) diversity patterns

**Fig. 3:**
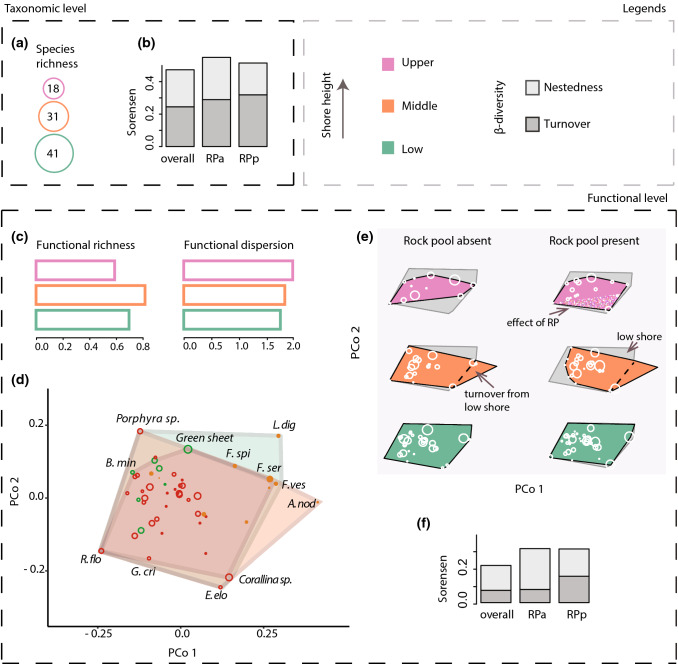
Seaweed functional ⍺- and β-diversity across intertidal zones (large scale), separated between the taxonomic and functional level. Panels **a–b** show species richness across zones and taxonomic β-diversity (divided into nestedness and turnover; for all communities and those with and without rock pools—RP). Panel **c** shows indices of functional richness (scaled to the potential maximum; details in Methods) and functional dispersion at each zone (all quadrats together). Panel **d** contains a representation of the two-dimensional trait space of the species pool (*n* = 50; PCoA biplot), indicating the zones with a coloured polygon. Species abbreviations are shown for those at the edges of the trait space at each height (complete names are given in Fig. 4). Panel *e* shows the same functional space, but separately for each zone and habitat type. Circle sizes on **d** represent five frequency categories (< 10, < 20, < 30, < 40 and < 50), with circle colours referring to the algae group (brown algae: filled orange, green algae: hollow green, and red algae: hollow red); circle sizes on **e** are proportional to species local frequency. Nestedness is observed when a polygon is inside a larger one (represented in grey on panel **e**), while turnover is an expansion of a polygon (indicated in dashed line). Although we did not address β-diversity between habitats, we indicate the addition to the functional space with the presence of rock pools at the upper shore (pixelated pink). Barplots in panel **f** show the partition of functional β-diversity components, overall and for the two habitat categories

**Fig. 4:**
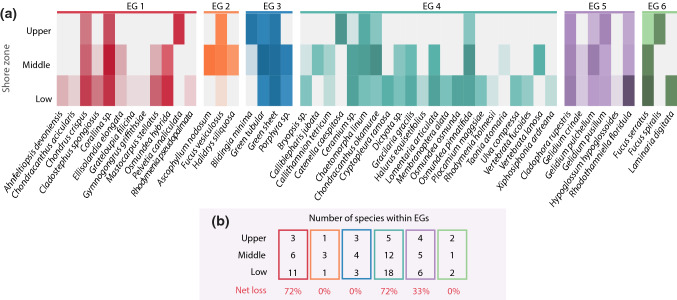
Species’ abundances and their assembly across the intertidal zones. **a** Species (*n* = 50) are sorted by emergent groups and abundance at each zone is log-transformed (values from 0—white—to 6—dark). **b** Number of species from each emergent group found at each zone, and percentage net loss from low to upper shore. Both panels are colour coded by emergent group (EG)

With increasing shore height, the number of species in each zone declined (Fig. 3a). Changes in the taxonomic composition between zones (β-diversity) were divided relatively evenly between nestedness and turnover, regardless of rock pool presence or absence (Fig. 3b). How this taxonomic loss and turnover translated into changes at the functional level depended on the metric. Functional richness peaked in the mid-shore, despite a continuous decline in species richness (Fig. 3c). Meanwhile, functional dispersion did not vary strongly across-shore levels, notwithstanding a slight increase towards higher zones (Fig. 3c). In addition to changes in the size of the functional space, there were also subtle changes in the relative location of the space, i.e. functional β-diversity (Fig. 3d–e). Unlike at the taxonomic level, these changes were dominated by nestedness rather than turnover (Fig. 3d–f). Indeed, the only observable functional turnover is with the expansion driven by *Ascophyllum nodosum* in the mid shore (Fig. 3e), while the rest of the changes are clearly via loss of parts of the trait space, especially in the absence of rock pools (Fig. 3f). Despite losses in functional richness, the presence of all emergent groups across zones illustrates the persistence of the overall ‘framework’ of functional space (Fig. 4a). A further look into emergent group richness between zones indicates that species loss was stronger within some groups than others (Fig. 4b), thus unevenly eroding functional redundancy. Overall, across zones, taxonomic diversity and composition changed more strongly than their functional counterparts.

### Small scale (quadrat level) diversity patterns

**Fig. 5:**
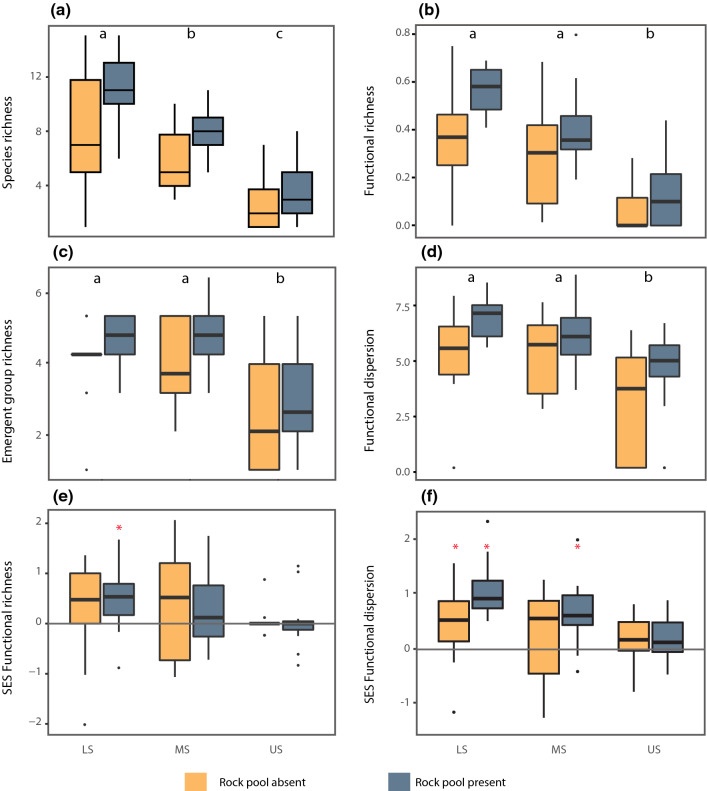
Observed and SES values of species’ and functional α-diversity. Boxplots show values across-shore zones (*LS* low shore, *MS* middle shore, and *US* upper shore), in quadrats with and without rock pools. Functional richness is scaled to the potential maximum (details in Methods). For observed values, different letters on top of boxes indicate zones are significantly different. For SES, values above or below the lines indicate deviance from null models, i.e. greater or lower than expected at random (asterisks indicate the significance of difference, obtained from one-sample *t* test, or Wilcoxon rank test when data distribution was not normal). Constrained randomizations are given in Fig. S6; SES without the zone-defining Phaeophytes are given in Fig. S1

Contrary to the zone scale, all α-diversity metrics decreased towards the higher shore at the small-scale (Table 2; Fig. 5a–d). Overall, the negative effect of height on diversity was stronger for species and functional richness than for emergent group richness and functional dispersion. Changes in local communities across zones were not constant: while species richness continuously decreased (Fig. 5a), the functional metrics only changed from middle to upper shore (Fig. 5b–d). Emergent group richness was strongly related to species richness (R^2^ = 0.83; see Appendix 5, ESM, for relationships between functional diversity metrics and species richness). Moreover, the presence of rock pools had an additive effect on all diversity metrics (Table 2). Generally, at the small scale, we observed further declines in functional diversity metrics with height, which were not evident at the large scale.

**Table 2:** Summary of main effects from (generalised) linear models ((G)LMs) with diversity measures (α-diversity) as responses, and shore zone, presence of rock pool (RP), and their interaction as predictors

GLMs	Deviance	Res. deviance	*F* value	*p* value
Species richness				
Zone	96.333	105.873	40.618	** < 0.001**
RP	13.117	92.756	11.061	** < 0.001**
Zone * RP	0.006	92.749	0.002	0.99
Emergent group richness				
Zone	14.373	33.648	18.411	** < 0.001**
RP	1.922	31.725	4.924	** < 0.05**
Zone * RP	0.124	31.601	0.159	0.85

LMs	Sum of squares	Mean SSq	*F* value	*p* value
Functional richness				
Zone	70,355	35,177	39.325	** < 0.001**
RP	11,249	11,249	12.575	** < 0.001**
Zone * RP	2327	1164	1.301	0.270
Functional dispersion				
Zone	94.670	47.340	13.955	** < 0.001**
RP	38.390	38.390	11.317	**0.001**
Zone * RP	2.470	1.240	0.365	0.69
SES Functional richness				
Zone	1.590	0.795	1.211	0.300
RP	0.110	0.115	0.175	0.680
Zone * RP	0.270	0.133	0.202	0.820
SES functional dispersion				
Zone	4.778	2.389	6.733	**0.002**
RP	2.546	2.546	7.174	**0.009**
Zone * RP	1.330	0.665	1.875	0.160

For species and emergent group richness we used generalised linear models (family: quasipoisson); for functional richness and dispersion we used general linear models. Significant values are in bold

*RPa* RP absent, *RPp*  RP present, *SES* standardised effect size

The standardised effect size (SES; comparing observed to null communities) showed different patterns for functional richness and dispersion (Table 2; Fig. 5e–f). Greater-than-expected values were observed for functional richness at the low shore with rock pools (Fig. 5e) and for functional dispersion in both low shore communities and middle shore with rock pools (Fig. 5f). The presence of rock pools had an additive effect on the SES of functional dispersion (*F* = 7.174, *p* < 0.01) but not on the SES of functional richness (*F* = 0.175, *p* = 0.7; Table 2). After obtaining these results, we ran the null models again, constraining the randomisations within zones, so that we could isolate the local effects from the whole-intertidal gradient effects. The over-dispersion was still observed, with patterns largely unchanged (Fig. S6, Appendix 6), indicating the role of small-scale (local) species interactions in driving functional over-dispersion. Overall, in low shore communities, species exhibited a greater total spread in trait space (functional richness) and abundant species were more distant to the centroid (functional dispersion) than expected, and this was further boosted in environments with rock pools. Contrary to our hypothesis there was no evidence that functional diversity was being limited due to environmental filtering at the upper shore (no *under*-dispersion).

## Discussion

Our results show how the species and functional diversity of seaweed assemblages—as an example of marine habitat formers—change over the intertidal gradient. With the loss and turnover of species across the gradient, functional diversity declines in a metric- and scale-dependent fashion. This study demonstrates the use of modern functional diversity approaches in seaweed assemblages and illustrates how functional diversity can deepen our understanding of changes in marine habitat former communities under environmental stress.

Consistent with expectations, functional diversity decreased with increasing shore height, although metric- and scale-dependently. Functional diversity often varies as a function of the facets and spatial scale considered, which may yield varying responses to environmental gradients (Smith et al. 2013). At the large scale, functional richness was the only metric to respond, illustrating its greater sensitivity to species-level changes and reflecting a decline in species with extreme functional traits in the upper shore. Further, the persistence of all emergent functional groups in the face of species loss can be attributed to the presence of redundant species within each group; although it is notable that certain emergent groups and upper shore assemblages have low redundancy and are thus vulnerable to future species loss. The finding that upper shore communities are nested within lower shore communities, rather than occupying a different part of trait space, illustrates a maintenance of the “core” functional structure, despite declines in functional richness. Together, the functional metrics reveal that, at large scales, the upper shore assemblages contain less functionally contrasting species, but retain a similar functional framework.

At the small scale, all metrics responded to the height gradient, although more strongly from middle to upper shore. For functional and emergent group richness, this pattern—changes only from middle to upper shore—can be explained by their non-linear responses to species richness (Appendix 5). From middle to upper shore, where species richness reaches low levels at the small scale, declines in richness are occurring closer to the first—steep—section of the curve (few species). In contrast, from the lower to middle shore, declines in species richness take place further along the curves, so they cause smaller changes in functional and emergent group richness. However, the explanation for the similar response of functional dispersion is not as simple because, in theory, this metric is invariant to species richness (Laliberté and Legendre 2010). Besides a species richness mediated scale-dependency, the scale is also expected to determine the relative importance of environmental filtering and biotic interactions (Smith et al. 2013). Thus, species interactions may also help explain scale-dependent functional diversity changes across the intertidal gradient.

Indeed, functional over-dispersion points towards a key role of species interactions in the assembly of functionally diverse seaweeds. In particular, two biotic mechanisms may be largely responsible. First, interspecific competition, which has been widely documented in seaweeds (Hawkins and Hartnoll 1985; Edwards and Connell 2012), may result in the exclusion of functionally similar species from local communities (Macarthur and Levins 1967; Adler et al. 2013), promoting dissimilarity in traits of co-occurring species. The stronger over-dispersion in the more favourable lower shore further supports a key role of competition (Bertness and Callaway 1994) and is consistent with a study using categorical traits across seaweeds and invertebrates on Chilean rocky shores (Valdivia et al. 2017). Second, positive interactions such as facilitation and associational defences could also support species with contrasting traits (Teagle et al. 2017). The co-occurrence of functionally distinct species may be further promoted by small-scale environmental heterogeneity, as indicated by the enhancement of functional diversity metrics in the presence of rock pools. Overall, it seems that local-scale processes—likely involving species interactions—drove over-dispersion and effectively boosted functional diversity in the low shore and may help explain scale-dependent functional diversity responses to shore height. Our finding that over-dispersion patterns were maintained even when null models were restricted to species occurring within each respective zone further strengthens this conclusion. Nevertheless, although observational studies such as ours describe naturally-occurring patterns of functional diversity and can show where functional diversity deviates from that expected under random assembly, only experiments can attempt to pin-down the relative and individual roles of the environment and species interactions in driving these patterns (Adler et al. 2013; Kraft et al. 2015).

Functional diversity approaches directly address the trait differences between species and, therefore, provide more insight than species-based measures into the potential consequences for ecosystem functioning (e.g. Le Bagousse-Pinguet et al. 2019). In our study, at the small scale, declines in functional richness and dispersion with increasing shore height reflect a reduction in both the total range of available traits and the distinctness of abundant species, potentially diminishing niche complementarity between co-occurring species (Cardinale et al. 2007; Stachowicz et al. 2008; Gamfeldt et al. 2015) and consequently ecosystem functioning (Griffin et al. 2009; Cadotte et al. 2011; Cadotte 2017). At the large scale, the smaller and nested functional space of upper shore communities, together with their lower redundancy, collectively indicate that these communities exhibit a reduced capacity to exploit environmental niches (Cardinale et al. 2000) and an impaired ability to withstand environmental changes (Loreau et al. 2003). Nevertheless, the persistence at this large scale of both functional dispersion and emergent group richness, as well as the maintenance of subset of core trait space, suggests resistance of some aspects of ecosystem functioning to the environmental gradient.

In conclusion, our results show that distinct aspects of seaweed functional diversity decline alongside species richness across the intertidal gradient. Yet patterns of change in functional diversity are scale-dependent: declines are stronger at the smaller scale, where both lower species richness and species interactions appear to be shaping functional diversity. These results bring a new perspective to the well-studied pattern of seaweed intertidal zonation while pointing towards the role of local, biotic factors in shaping seaweed community assembly and resulting functional diversity. Our study also demonstrates the importance of considering different spatial scales in studies of changes in biodiversity across communities (Chase et al. 2018). Functional diversity studies in marine species have traditionally been limited by a lack of appropriate trait data. However, these are now emerging for corals (Madin et al. 2016), seagrasses (de los Santos et al. 2016) and seaweeds (Mauffrey et al. 2020b). Applying these newly available functional traits to assemblages of marine habitat formers across temporal—or as we did, spatial—gradients allows the characterisation of context-dependent functional structure (e.g., redundancy) as well as insights into potential sensitivity to environmental change and attendant species loss. Future studies could use similar approaches along gradients such as coastal development, ocean acidification (volcanic vents), and climate change-driven species loss or turnover (see Teixidó et al. 2018; Hall-Spencer et al. 2008; Wernberg et al. 2011; Pessarrodona et al. 2019).

## Electronic supplementary material

Below is the link to the electronic supplementary material.

Supplementary file1 (DOCX 540 kb)
