# Supplementary material for: Functional diversity of habitat formers declines scale-dependently across an environmental stress gradient
Source: Oecologia. 2020 Sep 7;194(1):135–49. doi: 10.1007/s00442-020-04746-1 (PMC7561580; doi:10.1007/s00442-020-04746-1)
Supplement: Supplementary file 1 — Supplementary file1 (DOCX 540 kb) [file 442_2020_4746_MOESM1_ESM.docx]

**Electronic Supplementary Material for: Functional** **diversity of marine habitat formers declines scale-dependently across an environmental stress gradient**

Laura Cappelatti*, Alizée R. L. Mauffrey, John N. Griffin

Biosciences Department, Wallace Building, Swansea University, SA2 8PP, Swansea, Wales, UK

*Corresponding author: lauracappelatti@gmail.com. Orcid: 0000-0001-8525-9190

**List of appendices**

*Appendix 1.* Sensitivity analyses of diversity metrics by excluding the zone-defining Phaeophytes. Includes Table S1 (Summary of main effects from the linear models with diversity metrics) and Fig. S1 (Standardised effect sizes - SES).

*Appendix 2.* Sampling effort across zones and habitats. Includes Fig. S2 (Species accumulation curves) and Table S2 (Observed and extrapolated species richness).

*Appendix 3.* Additional information regarding traits and PCoA: methods and trait correlations. Includes Table S3 (Pearson correlations (R) between all pairs of continuous traits), Table S4 (Correlation (R) between the first PCo axes and study traits), and Fig. S3 (Quality of the trait space with different numbers of PCo axes).

*Appendix 4.* Diversity metrics for each study site individually. Includes Fig. S4.

*Appendix 5.* Relationship between functional richness and species richness. Includes Fig. S5 (Relationships between functional diversity metrics and species richness).

*Appendix 6*. Results from the standardised effect sizes (SES) constrained within zones. Includes Figure S6 (SES from randomizations constrained within zones).

**Appendix 1. Sensitivity analyses of diversity metrics by excluding the zone-defining Phaeophytes**

To test any possible bias caused by the species we used to define the zones (dominant Phaeophytes; fucoids and kelp) on the diversity metrics, we ran additional tests removing these species from analyses. Results were largely consistent with the main analyses, i.e. we still observe an effect of zone on all metrics except the SES of Functional richness (Table S1; Fig. S1). However, one change is that the effect of rock pool on the observed functional richness in no longer significant (p = 0.06; Table S1). For the SES analyses, we observed additional deviance from expected for functional dispersion in the MS+RP-absent and the US communities; for functional richness, instead of LS+RP present, it is LS+RP-absent which shows deviance from null (Fig. S1). Despite these small differences, since we are describing whole communities (including the dominant Phaeophytes) these results should only serve as an indication that the non-expected patterns described in the main text are not strongly biased by the zone-defining species.

**Table S1. Summary of main effects from (generalised) linear models ((G)LMs) with diversity dimensions (⍺-diversity) without zone-defining Phaeophytes, and shore zone and presence of rock pool (RP).** For species and emergent group richness we used generalised linear models (family: quasipoisson); for functional richness and dispersion we used general linear models. Significant values are in bold. RPa = RP absent; RPp = RP present.

| GLMs | **Deviance** | **Res. deviance** | **F value** | **P value** |
| --- | --- | --- | --- | --- |
| *Species richness* |  |  |  |  |
| Zone | 66.955 | 96.078 | 28.299 | **<0.001** |
| RP | 10.043 | 86.034 | 8.489 | **<0.001** |
| Zone * RP | 0.118 | 85.916 | 0.050 | 0.95 |
| *Emergent group richness* |  |  |  |  |
| Zone | 8.373 | 21.05 | 16.942 | **<0.001** |
| RP | 1.463 | 19.587 | 5.920 | **0.017** |
| Zone * RP | 0.215 | 19.371 | 0.436 | 0.648 |
| LMs | **Sum of Squares** | **Mean SSq** | **F value** | **P value** |
| *Functional richness* |  |  |  |  |
| Zone | 51.25 | 25.626 | 5.523 | **0.006** |
| RP | 16.59 | 16.588 | 3.575 | 0.063 |
| Zone * RP | 10.26 | 5.129 | 1.105 | 0.337 |
| *Functional dispersion* |  |  |  |  |
| Zone | 3.276 | 1.638 | 4.015 | **0.022** |
| RP | 2.526 | 2.526 | 6.193 | **0.015** |
| Zone * RP | 0.31 | 0.155 | 0.38 | 0.685 |
| *SES Functional richness* |  |  |  |  |
| Zone | 2.31 | 1.155 | 1.55 | 0.219 |
| RP | 0.00 | 0.001 | 0.002 | 0.965 |
| Zone * RP | 2.56 | 1.280 | 1.717 | 0.187 |
| *SES Functional dispersion* |  |  |  |  |
| Zone | 10.57 | 5.287 | 3.002 | **0.056** |
| RP | 6.76 | 6.761 | 3.839 | **0.054** |
| Zone * RP | 0.33 | 0.167 | 0.095 | 0.909 |


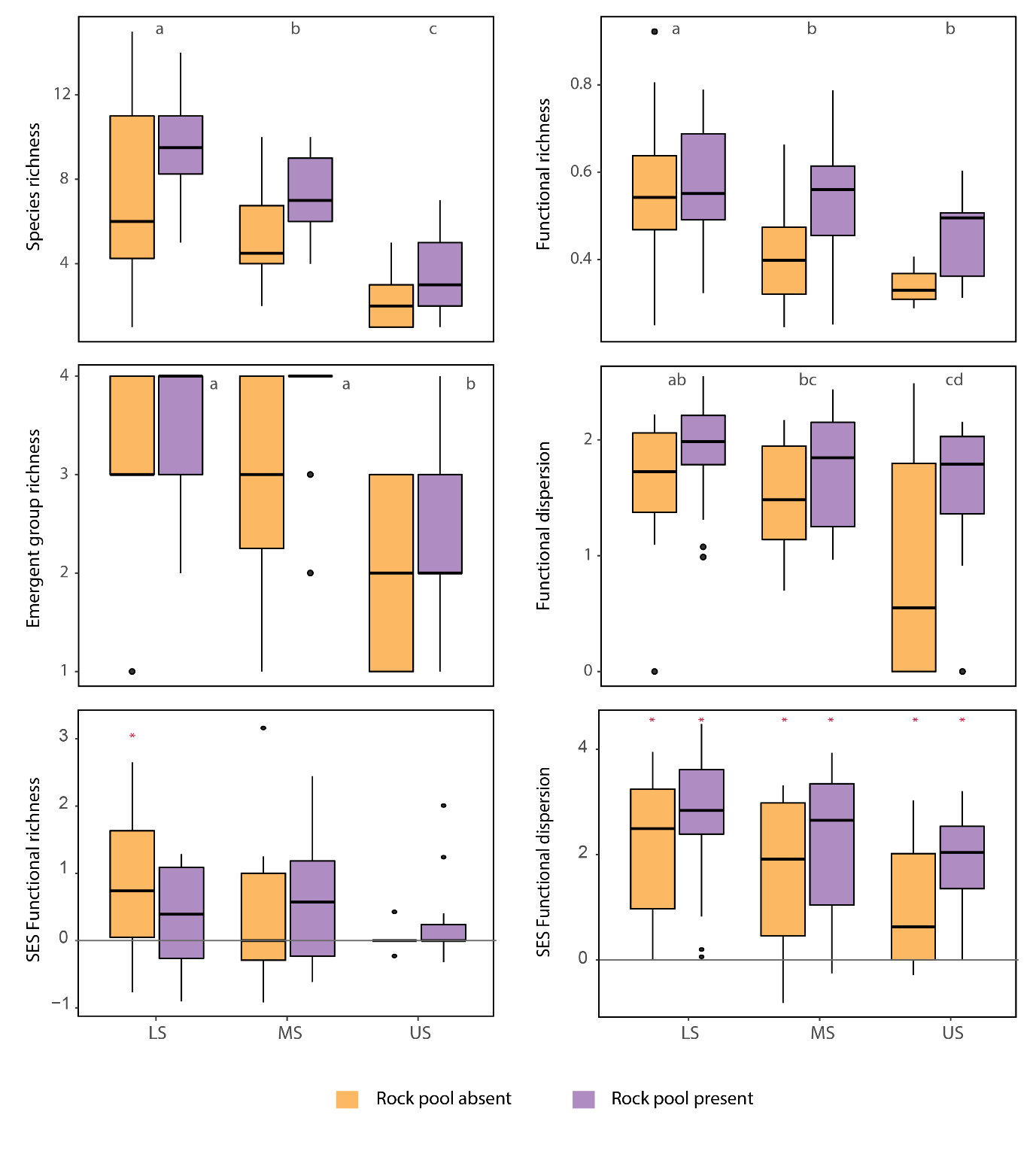


**Fig. S1. Observed and SES values of species’ and functional ⍺-diversity without zone-defining Phaeophytes.** Boxplots show values across shore zones (LS = low shore, MS = middle shore and US = upper shore), in quadrats with and without rock pools. Functional richness is scaled to the potential maximum (details in Methods). For observed values, different letters on top of boxes indicate zones are significantly different. For SES, values above or below the lines indicate deviance from null models, i.e. greater or lower than expected at random (asterisks indicate significance of difference, obtained from one-sample t test, or Wilcoxon rank test when data distribution was not normal).

**Appendix 2. Sampling effort across zones and habitats.**

After randomly selecting quadrats to have an equal representation of zone and habitat type (n=84), to test if we adequately sampled the three shore zones among the two habitat categories, we plotted species accumulation curves and extrapolated species richness per community (Chao1 index). We used functions *specaccum* and *specpool*, respectively, on *vegan* package (Oksanen et al 2019). Accumulation curves started to stabilize, but did not fully reach an asymptote, indicating that more sampling would still add to the list of species (Fig. S2). In general, the richness in the upper shore was better sampled (Table S2), and at least 79% of each community was estimated, compared to the extrapolated richness values. Despite the small underestimation of the lower/middle shore, we still observed higher alpha diversity and SES values in the lower zones, therefore we can be safe that these results are not a product of a sampling bias – at worst they are an underestimation of low-shore richness and diversity.


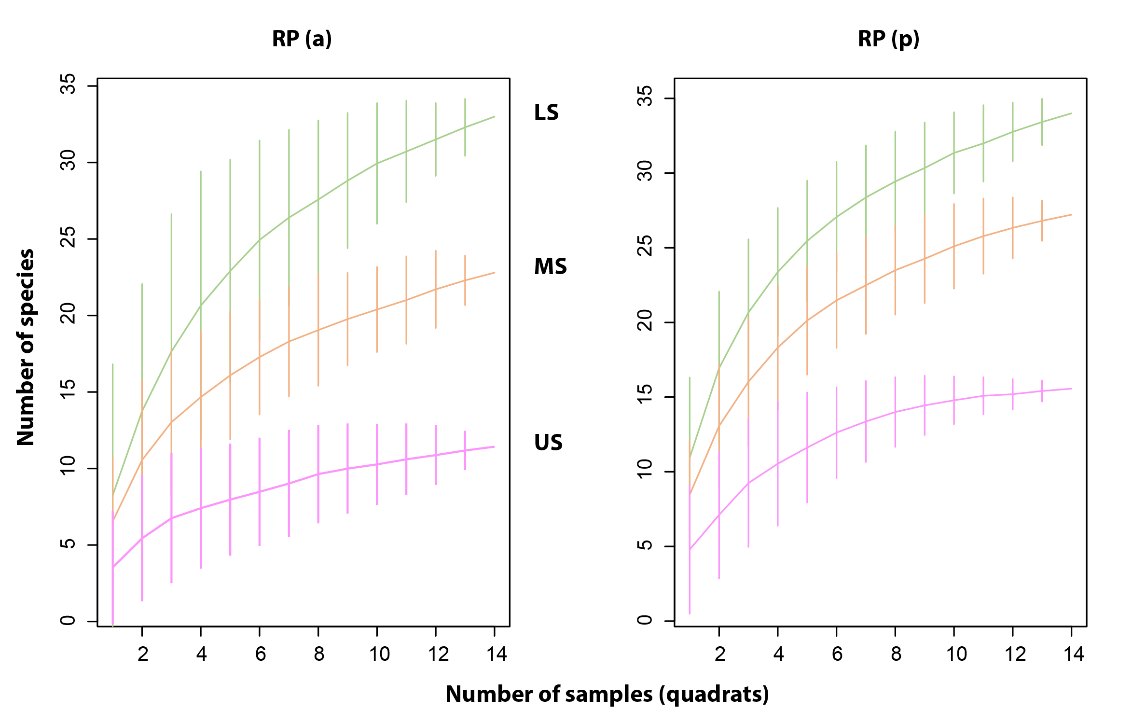


**Fig. S2.** **Species accumulation curves for the three shore zones (LS=low shore, MS=middle shore and US=upper shore), for the two habitat categories (RP(a)=rock pool absent and RP(p)=rock pool present).**

**Table S2. Observed and extrapolated species richness.** Number of species (incidence-based) with method Chao, by each zone overall and among habitat categories (RP (a) = rock pool-absent, RP (p) = rock pool-present). % = how much of estimated richness was sampled, in percentage.

| Community | Category | Low shore | Middle shore | Upper shore |
| --- | --- | --- | --- | --- |
| Observed | overall | 41 | 31 | 18 |
| Extrapolated |  | 49 | 37 | 19 |
| % |  | 84 | 84 | 95 |
| Observed | RP (a) | 33 | 23 | 11 |
| Extrapolated |  | 39 | 30 | 18 |
| % |  | 85 | 77 | 61 |
| Observed | RP (p) | 34 | 27 | 15 |
| Extrapolated |  | 46 | 37 | 15 |
| % |  | 74 | 73 | 100 |

**Appendix 3. Additional information regarding traits and Principal coordinate analysis.**

*List of traits:* a) surface area to volume ratio (SA:V); b) specific thallus area (STA); c) surface area to perimeter ratio (SA:P); d) thallus dry matter content (TDMC); e) thickness; f) length; g) branching order, h) Carbon to Nitrogen ratio (C:N).

*Trait measurement methods*: To obtain specific thallus area, for traits *a - c*, we scanned or photographed the fresh, clean samples. Individuals were scanned on an Epson Perfection V600, V39, (Suwa, Japan) or photographed directly (Pentax K3 digital camera, SMC DA L 18-55 mm, Tokyo, Japan) on a lightbox (MiniSun A1, Manchester, UK). Finer samples were photographed with an imaging microscope (Leica S8AP0, Wetzlar, Germany, affixed with GT Vision GXCAM-H3, Sudbury, UK). Some species have no distinction of thallus parts (holdfast, stipe, and fronds), in which case they were considered in their entirety (thallus), otherwise we took the area of the fronds. Area and perimeter of samples were calculated using the software ImageJ (Schneider, Rasband, & Eliceiri, 2012). Surface area was multiplied by two, as both sides are photosynthetically active.

To obtain dry mass needed for traits *b* and *d*, we cleaned and oven-dried the respective fronds/thalli for a week. For *e*, we used an electronic thickness gauge (Digital Micrometers Ltd, DTG03 0.005, DML3032 0.001 mm, Sheffield, UK); some species are very fine and delicate, so we measured their thickness under the microscope, with a micrometric scale. For *f* we used a tape measure or the microscope. *g* was obtained by counting the number of times the main axis of the thallus divided in two and then calculating the average of five different measurements. Carbon and Nitrogen for *h* were measured on dry ground samples in an elemental analyser (PDZ Europa 2020 isotope ratio mass spectrometer interfaced with an ANCA GSL elemental analyser and calibrated with acetanilide).

**Table S3.** Pearson correlations (R) between all pairs of continuous traits.

|  | SAV | STA | SA:P | TDMC | Thick | Length | Branch | C:N |
| --- | --- | --- | --- | --- | --- | --- | --- | --- |
| SAV | 1 | 0.8 | -0.22 | 0.12 | -0.75 | -0.39 | -0.18 | -0.47 |
| STA |  | 1 | -0.06 | -0.25 | -0.7 | -0.3 | -0.23 | -0.61 |
| SA:P |  |  | 1 | -0.23 | 0.17 | 0.52 | -0.21 | 0.29 |
| TDMC |  |  |  | 1 | -0.16 | -0.19 | 0.06 | -0.27 |
| Thick |  |  |  |  | 1 | 0.48 | 0.1 | 0.46 |
| Length |  |  |  |  |  | 1 | -0.31 | 0.51 |
| Branch |  |  |  |  |  |  | 1 | 0.15 |
| C:N |  |  |  |  |  |  |  | 1 |

Details on the PCoA and its derived metrics

The functional space of our study species was reduced via a Gower distance-based PCoA (dbFD; Villéger et al. 2008). We then used the first two dimensions of the PCoA to calculate community-level indices of functional richness and dispersion. To test the quality of the two-dimensional representation of the functional space, we used a function described and created by Maire et al. (2015), which computes the mean squared deviation between the initial functional distance (in this case, Gower) and the standardised distance in the functional space, based on functional dendrograms. It is then possible to choose from several dimensions. Although more dimensions are better, we confirmed that the first two dimensions are in congruence with the initial distance (Fig. S3) and therefore a good representation of the functional space among our study species.


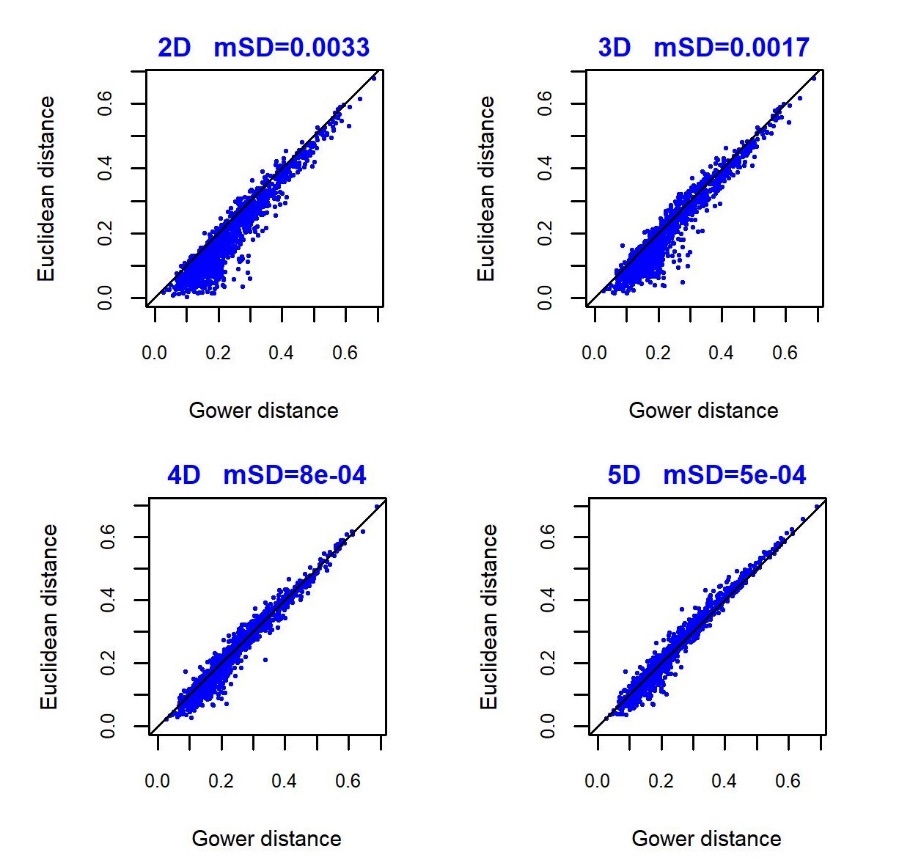


**Fig. S3. Graphical representation of the quality of functional representation for two to five dimensions.** Panels illustrate quality of each n-dimensional functional space built from a principal coordinate analysis, showing correlation of species pairs between initial (Gower) and reduced (Euclidean) functional distances.

At the small scale, only PCo axes 1-2 were kept to meet the requirement of species > traits per community; at the large scale we included one more axis to more faithfully represent the underlying trait variation among species (PCo axes 1-2 and 1-3 explained 60.9% and 71.6% of inertia). Note that sampling curves (Fig. S2) indicate that we underestimated total zone-level species richness. The upper zone is closer to estimated asymptotic richness levels than the low or middle zones, indicating that declines in species richness and functional richness (sensitive to species richness) between lower zones and the upper zone are likely to be conservative estimates. Importantly, however, qualitative richness differences across zones were not scale-dependent (the sample-based species accumulation curves do not overlap).

**Table S4.** **Correlation (R) between the first three PCo axes and study traits.** ANOVA was used for the categorical trait pneumatocysts (abbreviated as “Pneum.”). Strongest associations are highlighted in bold and negative correlations in red.

| PCo axis | STA | SAV | TDMC | Thickness | Length | SAP | Branching | C:N | Pneum. |
| --- | --- | --- | --- | --- | --- | --- | --- | --- | --- |
| 1 | **-0.85** | **-0.83** | 0.04 | **0.81** | **0.64** | 0.35 | 0.13 | **0.79** | **0.43** |
| 2 | 0.34 | -0.01 | **-0.79** | 0.06 | 0.51 | **0.61** | **-0.42** | -0.18 | 0 |
| 3 | -0.16 | -0.39 | **-0.48** | 0.35 | -0.29 | -0.36 | **0.41** | -0.36 | 0 |

**Appendix 4. Diversity metrics for each study site individually**.

**
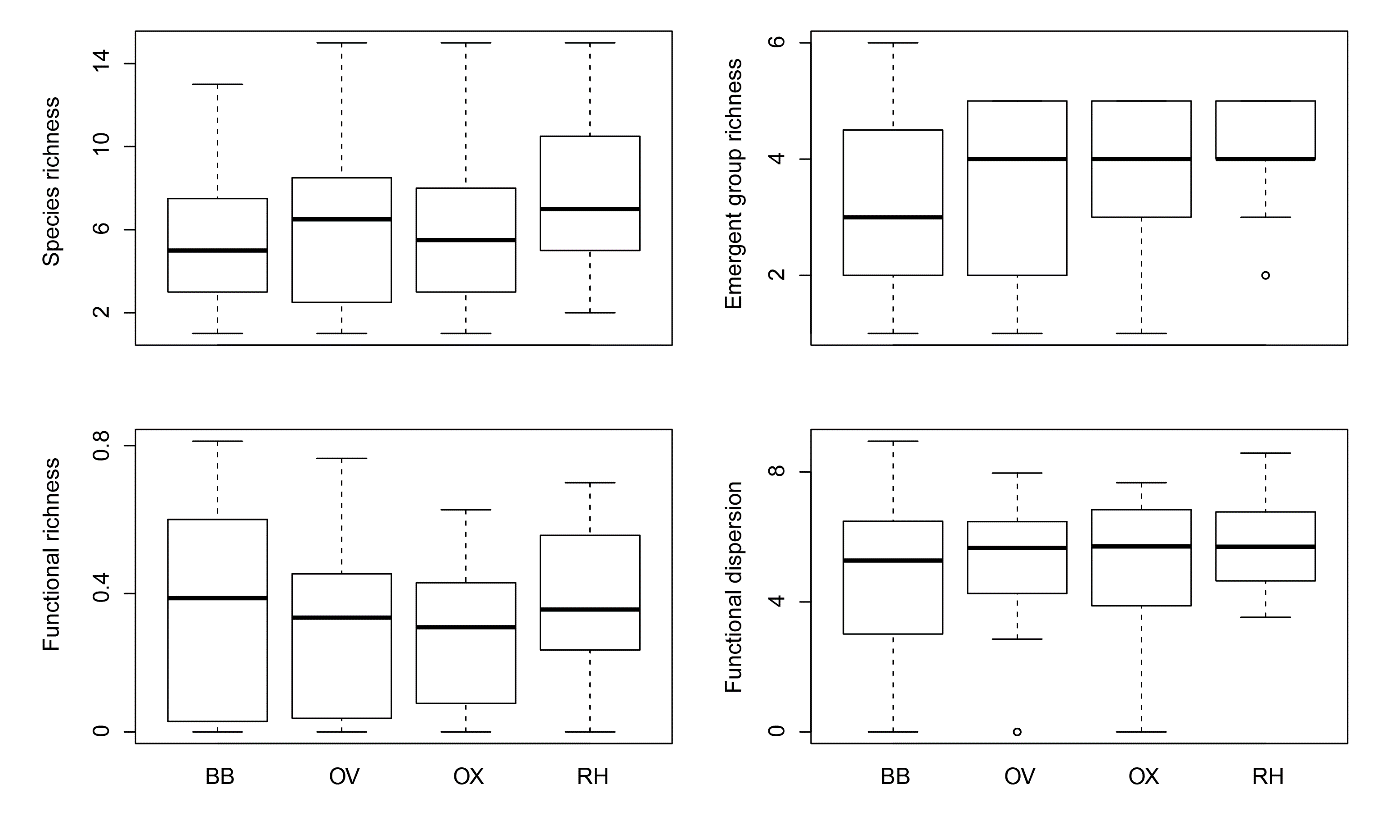
**

**Fig. S4.** **Comparing diversity metrics across sites.** Diversity metrics (alpha diversity) measured across all study sites in the Gower peninsula, Wales, UK. Functional richness is scaled to potential maximum (see Methods in main). BB= Bracelet Bay; OV= Overton; OX= Oxwich; RH= Rhossili.

**Appendix 5. Relationship between functional richness and species richness**.


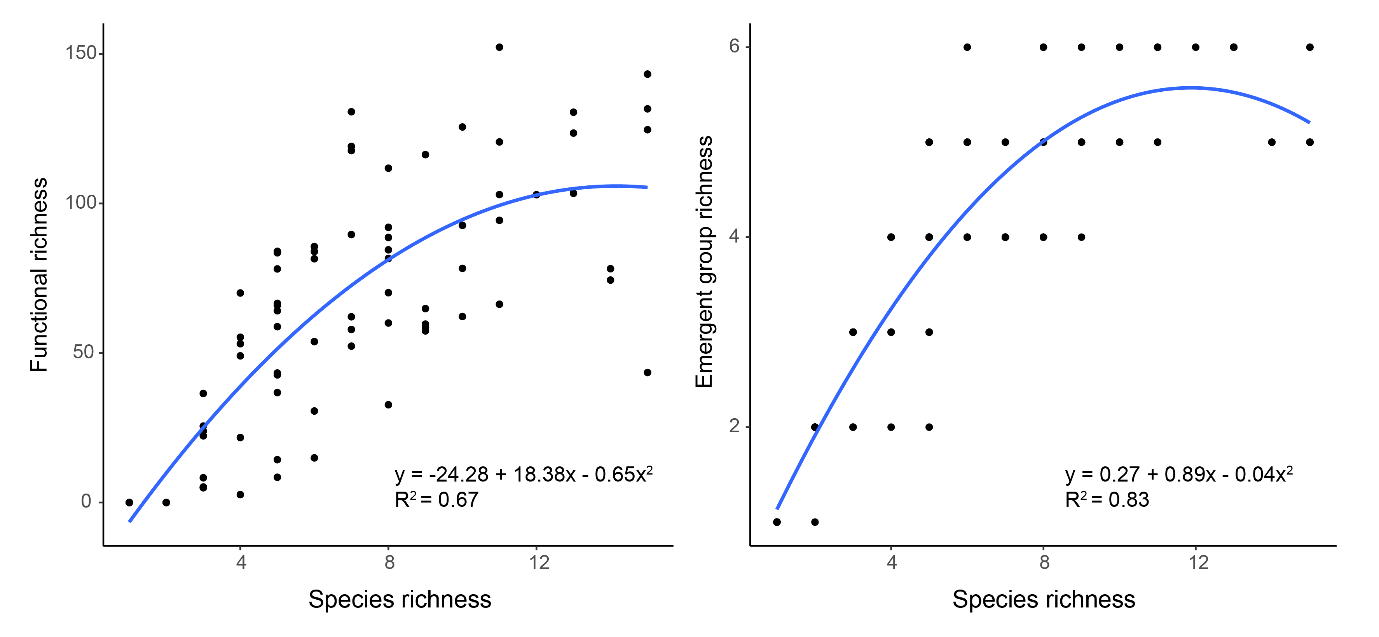


**Fig. S5.** **Relationships (quadratic regression) between functional richness and species richness, and correlation between emergent group richness and species richness, across intertidal macroalgae communities.**

**Appendix 6. Results from standardised effect sizes (SES) constrained within zones**.

We ran additional null models to isolate the effect of factors within zones from the large effect of the intertidal gradient. We did that by constraining randomizations within species found in each zone. Results were consistent with the main SES tests (Fig. S6).

**
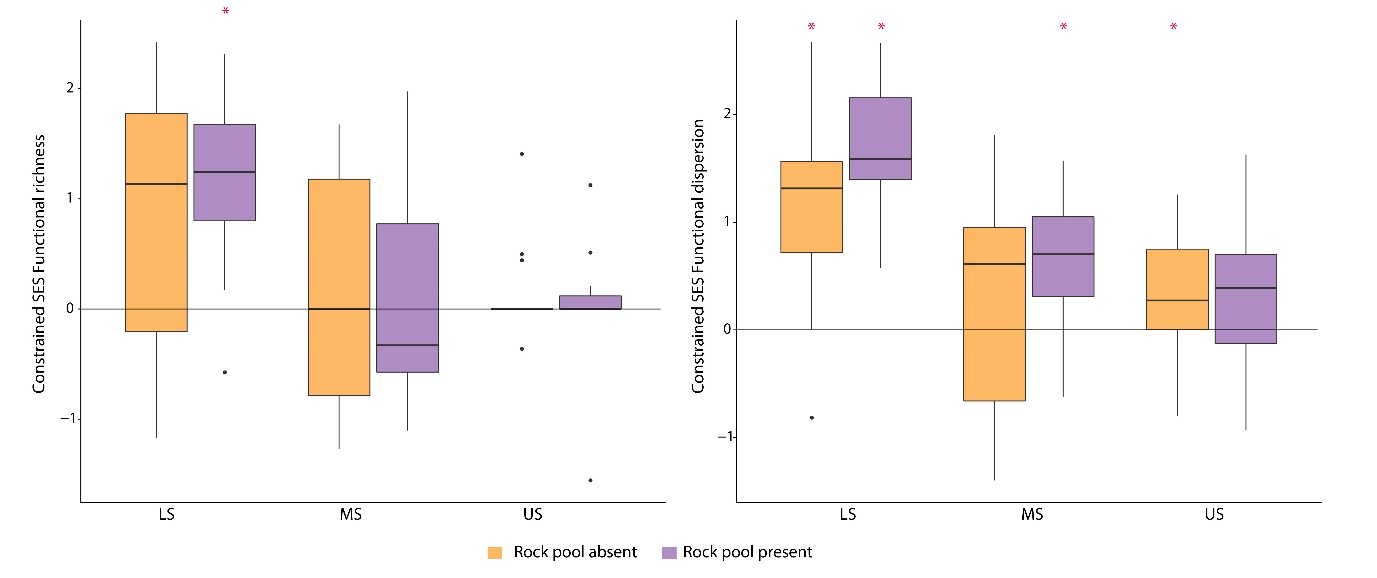
**

**Fig. S6.** **Standardised effect sizes (SES) from randomizations constrained within zones.** Boxplots show SES values of functional dispersion across shore zones (LS=low shore, MS=middle shore and US=upper shore). Values not touching the lines indicate deviance from null models, i.e. above or below expected at random. Asterisks indicate significant difference from zero (obtained from one-sample t test, or Wilcoxon rank test when data distribution was not normal).

**References**

Maire E, Grenouillet G, Brosse S, Villéger S (2015). How many dimensions are needed to accurately assess functional diversity? A pragmatic approach for assessing the quality of functional spaces: Assessing functional space quality. Global Ecology and Biogeography 24:728–740. doi: 10.1111/geb.12299

Oksanen J et al (2019). vegan: Community Ecology Package. R package version 2.5-4. https://CRAN.R-project.org/package=vegan

Schneider C A, Rasband WS, Eliceiri KW (2012). NIH Image to ImageJ: 25 years of image analysis. Nature Methods, 9(7), 671–675. doi: 10.1038/nmeth.2089.

Villéger S, Mason NWH, Mouillot D (2008) New Multidimensional Functional Diversity Indices for a Multifaceted Framework in Functional Ecology. Ecology 89:2290–2301
